# Achieving descriptive accuracy in explanations *via* argumentation: The case of probabilistic classifiers

**DOI:** 10.3389/frai.2023.1099407

**Published:** 2023-04-06

**Authors:** Emanuele Albini, Antonio Rago, Pietro Baroni, Francesca Toni

**Affiliations:** ^1^Department of Computing, Imperial College London, London, United Kingdom; ^2^Dipartimento di Ingegneria dell'Informazione, Università degli Studi di Brescia, Brescia, Italy

**Keywords:** argumentation, descriptive accuracy, explainable AI, probabilistic classifiers, properties

## Abstract

The pursuit of trust in and fairness of AI systems in order to enable human-centric goals has been gathering pace of late, often supported by the use of *explanations* for the outputs of these systems. Several properties of explanations have been highlighted as critical for achieving trustworthy and fair AI systems, but one that has thus far been overlooked is that of *descriptive accuracy* (DA), i.e., that the explanation contents are in correspondence with the internal working of the explained system. Indeed, the violation of this core property would lead to the paradoxical situation of systems producing explanations which are not suitably related to how the system actually works: clearly this may hinder user trust. Further, if explanations violate DA then they can be deceitful, resulting in an unfair behavior toward the users. Crucial as the DA property appears to be, it has been somehow overlooked in the XAI literature to date. To address this problem, we consider the questions of formalizing DA and of analyzing its satisfaction by explanation methods. We provide formal definitions of *naive, structural* and *dialectical* DA, using the family of probabilistic classifiers as the context for our analysis. We evaluate the satisfaction of our given notions of DA by several explanation methods, amounting to two popular feature-attribution methods from the literature, variants thereof and a novel form of explanation that we propose. We conduct experiments with a varied selection of concrete probabilistic classifiers and highlight the importance, with a user study, of our most demanding notion of dialectical DA, which our novel method satisfies by design and others may violate. We thus demonstrate how DA could be a critical component in achieving trustworthy and fair systems, in line with the principles of human-centric AI.

## 1. Introduction

Equipping automated decision systems with explanation capabilities is a compelling need which lies at the basis of the rapid growth of the research field of explainable AI (XAI) in recent years (Guidotti et al., 2019) and is receiving an increasing attention from government and regulatory bodies, like the European Commission. Quoting the report of the Independent high-level expert group on Artificial Intelligence set up by the European Commission (2019): “Whenever an AI system has a significant impact on people's lives, it should be possible to demand a suitable explanation of the AI system's decision-making process. Such explanation should be timely and adapted to the expertise of the stakeholder concerned (e.g., layperson, regulator or researcher).”

By providing explanations, a system goes beyond just presenting its outcomes as oracles: rather, they are subjected to the scrutiny of the cognitive capabilities of the users, who receive means to achieve a better understanding of the reasons underlying system's decisions and/or of its internal operation. In this way, the adoption of an active and conscious role by users is supported: they will be able to criticize or agree with system decisions, based on a cognitively elaborated motivation, rather than blindly rejecting or accepting them. Thus, explanations for the outputs of AI systems are widely understood as crucial to support trust in these systems (Ribeiro et al., 2016; Miller, 2019; Zerilli et al., 2022).

Due to their role in promoting users' understanding and involvement, it is no surprise that the two properties of *cognitive explainability and transparency* are widely regarded as key factors and technical challenges of Human-Centric AI, as evidenced in the introductory article of this special issue (Kakas et al., 2022). For instance, “Make clear why the system did what it did” is one of the design guidelines for human-AI interaction presented by Amershi et al. (2019), while the Research Roadmap of the European network of Human-Centered Artificial Intelligence (www.humane-ai.eu) regards the fact that AI systems are explainable and accountable as a basic prerequisite for human-in-the-loop activities.

This paper contributes to the development of explainability for human-centric AI by proposing a formal treatment of the notion of *descriptive accuracy* (DA), a crucial property for explanations supporting fair AI systems deserving trust, and by showing how DA requirements can be achieved in practice through a suitable form of explanation, called *DARXs* (acronym for *Dialectically Accurate Relational Explanations*). Both the formal treatment of DA and the definition of DARX are based on ideas and formalisms from the field of *Argumentation Theory*, connecting the present contribution to the subject of the special issue. Argumentation theory (also referred to in the literature as *computational* or *artificial* argumentation, e.g., see Atkinson et al., 2017; Baroni et al., 2018 for overviews) has recently been advocated, in a variety of ways, as a mechanism for supporting explainable AI (see Cyras et al., 2021; Vassiliades et al., 2021 for recent surveys). A popular use thereof is as a means for representing the information in an existing AI system in a way which is more amenable for human consumption than typical explanation methods, e.g., as in Timmer et al. (2015) and Rago et al. (2021). This use of argumentation is the inspiration also for this paper: the formulations of DA we propose are defined for abstract notions of explanation inspired by the argumentation frameworks in the seminal works of Toulmin (1958) and Cayrol and Lagasquie-Schiex (2005), DARXs are inspired by bipolar argumentation frameworks (Cayrol and Lagasquie-Schiex, 2005); and our definitions of DA bear resemblance to properties originally proposed (for various forms of argumentation frameworks) by Amgoud and Ben-Naim (2018) and by Baroni et al. (2019).

The paper is organized as follows. Section 2 presents the motivations and contribution of the work, in particular positioning our contribution in the context of the special issue, while Section 3 discusses related works. Then, after providing the required preliminary notions in Section 4, we introduce the proposed formal treatment of DA in Section 5. Section 6 examines the satisfaction of DA by some existing and novel explanation approaches, showing that, differently from other proposals, DARX guarantees a full satisfaction of DA requirements. These formal results are backed by an experimental evaluation in Section 7 and by a human experiment in Section 8, before concluding in Section 9.

## 2. Motivations and contribution

Being immersed in the human-centric perspective, the issue of realizing explainable and transparent system does not only represent a challenging and fascinating socio-technical problem to tackle (Miller, 2019), but also involves substantial ethical aspects and requires the satisfaction of human-centric properties, like trustworthiness and fairness.

First, the explanations provided for the outputs of a system are a key factor in achieving user *trust*, a prerequisite for acceptance of the decisions of a system when deemed to be trustworthy. However, as pointed out by Jacovi et al. (2021), trust, which is an attitude of the trustors (in our case, the systems' users), is distinguished from trustworthiness, which is a property of the trustees (in our case the explained systems), i.e., the capability of maintaining some *contract* with the users. In fact, “trust and trustworthiness are entirely disentangled: … trust can exist in a model that is not trustworthy, and a trustworthy model does not necessarily gain trust” (Jacovi et al., 2021). This makes the goal of achieving trust, and the role of explanations therefor, a rather tricky issue. On the one hand, there can be situations where trust is achieved by explanations which are convincing but somehow deceptive. On the other hand, there can be situations where an otherwise trustworthy system loses users' trust due to problems in its explanations' capabilities, e.g., as pointed out by Jacovi et al. (2021), For illustration, consider the case of an AI-based medical system predicting, for a patient, a high risk of getting disease X and including in the explanation the fact that some parameter Y in the patient's blood test is high. If the patient deems the system trustworthy, they may try to change (if possible) the value of Y, e.g., by lifestyle changes. If they find out that the value of Y was actually irrelevant, i.e., the diagnosis would have been the same with a low value of Y, and thus trying to modify it will not have the intended impact on the system prediction, then the patient's trust will be negatively affected, independently of the correctness of the diagnosis.

Thus, trust in an otherwise accurate system can be hindered or even destroyed by some drawbacks of the explanations it provides.

Trust is however not the only issue at stake. Continuing the illustration, suppose the patient never gets to know about the irrelevance of parameter Y. Then, their trust may be preserved, but then a possibly deceitful system would remain in place. This shows that, in connection to their impact on trust, explanations also have an important role toward *fairness* of AI systems: the description of the principle of *fairness* in the report by the Independent high-level expert group on Artificial Intelligence set up by the European Commission (2019) states that “the use of AI systems should never lead to people being deceived or unjustifiably impaired in their freedom of choice.” This indication complements the requirement of “ensuring equal and just distribution of both benefits and costs, and ensuring that individuals and groups are free from unfair bias, discrimination and stigmatization.” Two complementary facets of fairness emerge here. The latter concerns a possible unjust treatment caused by system biases toward specific user features, while the former addresses the risk that the system may treat its users improperly due to inappropriate design choices for the explanations. This form of unfairness applies to *all* users, rather than just *some*, as in the case of selective system biases.

Avoiding *selectively unfair* (biased) systems is receiving a great deal of attention in the literature (see, for instance, Dwork et al., 2012; Heidari et al., 2019; Hutchinson and Mitchell, 2019; Binns, 2020; Räz, 2021), whereas the problem of avoiding *uniformly unfair* systems (due to ill-founded explanations) is receiving less attention, in spite of being no less important.

These considerations call for the need of identifying some basic formal requirements that explanations should satisfy in order to lead to (deservingly) trustworthy as well as (uniformly) fair AI systems. Indeed, providing a formal counterpart to these high-level principles appears to be crucial in order to carry out the following activities in a well-founded and non-ambiguous way: defining methods for quality verification and assurance from a human-centric perspective, comparing the adequacy of different systems on a uniform basis, providing guidelines for system development. Universal and absolute notions of trustworthiness and fairness being elusive, if not utopical, we share the suggestion that “the point is not complete fairness, but the need to establish metrics and thresholds for fairness that ensure trust in AI systems” (Dignum, 2021).

In turn, the investigation of formal requirements for explanations can benefit from a reference conceptual environment where their definition can be put in relation with some general foundational notions, whose suitability with respect to the human-centric perspective is well-established. Formal argumentation is an ideal candidate in this respect, for the reasons extensively illustrated in particular in Sections 3.1, 4.1 of Kakas et al. (2022) from which we limit ourselves to cite the emblematic statement that “Argumentation has a natural link to explanation.” Thus, it is not surprising that several works have focused on the use of argumentative techniques for a variety of explanation purposes (Cyras et al., 2021; Vassiliades et al., 2021). However, the study of argumentation-inspired formal properties related to human-centric issues like trustworthiness and fairness appears to have received lesser attention.

As a contribution to fill this gap, in this paper we use argumentation as a basis to formalize the property of *descriptive accuracy* (DA) described by Murdoch et al. (2019), for machine learning in general, as “the degree to which an interpretation method objectively captures the relationships learned by machine learning models.” DA appears to be a crucial requirement for any explanation: its absence would lead to the risk of misleading (if not deceptive) indications for the user (thus affecting trust and fairness). As such, one would expect that any explanation method is either able to enforce DA by construction or is equipped with a way to unearth possible violations of this fundamental property.

Specifically, we address the issue of defining argumentation-inspired formal counterparts (from simpler to more articulated) for the general notion of DA. In particular, our proposal leverages on two main sources from the argumentation literature: *Toulmin's argument model* (Toulmin, 1958) and the formalism of *bipolar argumentation frameworks* (Cayrol and Lagasquie-Schiex, 2005; Amgoud et al., 2008; Cayrol and Lagasquie-Schiex, 2013). In a nutshell, Toulmin's argument model focuses on providing patterns for analyzing argument structure at a conceptual level. The most fundamental argument structure consists of three elements: claim, data and warrant. The *claim* of an argument is the conclusion it brings forward; the *data* provide evidence and facts which are the grounds in support of the claim; and the *warrant*, which could be implicit, links the data to the claim. Bipolar argumentation frameworks belong to the family of abstract argumentation formalisms pioneered by Dung (1995), where arguments are seen as abstract entities, and the main focus is on the relations among arguments, their meaning, and their role in the assessment of argument status. In particular, bipolar argumentation encompasses the basic relations of *attack* and *support* which provide a synthetic and powerful abstraction of the main kinds of dialectical interactions that may occur between two entities (see, for instance, Tversky and Kahneman (1992) and Dubois et al. (2008) for general analyzes emphasizing the role of bipolarity in human decisions). A bipolar argumentation framework is hence a triple (*Args, Att, Supp*) where *Att, Supp*⊆*Args*×*Args*.

We will see that some of our abstractions for explanations can be put in correspondence with Toulmin's model with an implicit warrant, whereas others can be seen as bipolar argumentation frameworks. Argumentation frameworks are typically equipped with “semantics” (e.g., notions of extensions) that may satisfy desirable properties: we define notions of DA drawing inspiration from some of these properties.

On these bases, focusing on the setting of *probabilistic classifiers*, we make the following contributions.

- We introduce three formal notions of DA (Section 5): *naive* DA, as a precursor to *dialectical DA*, both applicable to any probabilistic classifier, and *structural DA*, applicable to probabilistic classifiers that are equipped with a *structural description*, as is the case for *Bayesian network classifiers* (BCs) (see Bielza and Larrañaga, 2014 for an overview) and *Chain Classifiers* (CCs), resulting from chaining probabilistic classifiers (e.g., as is done for BCs by Read et al., 2009 and for other types of probabilistic classifiers by Blazek and Lin, 2021). These notions of DA are defined for generic abstractions of explanations, so that they can be applied widely to a variety of concrete notions instantiating the abstractions.
- We study whether concrete explanation methods (instantiating our abstract notions of explanation) satisfy our notions of DA (Section 6). We focus our analysis on (i) existing *feature attribution methods* from the literature, namely LIME (Ribeiro et al., 2016) and SHAP (Lundberg and Lee, 2017), as well as (ii) novel variants thereof and (iii) a novel method we define (which we refer to in short as DARX). We prove that: the methods (i) are not guaranteed to satisfy any of the formulations of DA we define; the variants (ii) are guaranteed to satisfy structural DA (by construction) but may still violate (naive and) dialectical DA; the DARX method is guaranteed to satisfy all the considered forms of DA (by construction), thus providing a proof of concept that our forms of DA are indeed satisfiable.
- We evaluate our forms of DA empirically on a variety of BCs and CCs (Section 7),^1^ showing that they are often violated in practice by methods (i) and (ii).
- We describe a user study we conducted to gauge the importance of dialectical DA (our most “demanding” form of DA, applicable to any probabilistic classifier) to humans, when using explanations of probabilistic classifiers (Section 8), showing that this property predominantly aligns with human judgement.

## 3. Related work

A multitude of methods for providing explanations have been proposed (e.g., see the survey by Guidotti et al., 2019) and their desirable properties have been considered from a variety of perspectives (e.g., see the survey by Sokol and Flach, 2020). We draw inspiration from Murdoch et al. (2019) and focus, in particular, on their property of *descriptive accuracy* (DA) for (model-based or post-hoc) interpretable machine learning. As mentioned in the introduction, DA concerns the degree to which an interpretation (in our setting, explanation) method objectively captures the behavior of the machine-learned models. We will build on argumentative notions to provide three *formal* characterisations for DA, allowing evaluation of explanation methods for satisfaction of DA in precise terms.

DA is seen, in Murdoch et al. (2019), as a crucial property for achieving interpretable machine learning, alongside, in particular, *predictive accuracy*, wrt (test) data, of the predictions produced by the interpretations/explanations. Whereas DA is concerned with the inner workings of models, predictive accuracy is concerned with the input-output behavior thereof. Predictive accuracy is thus closely linked with properties of *fidelity* or *faithfulness* which have been considered by several works. For instance, in Guidotti et al. (2019) fidelity is defined as the capability of an explanation model to “accurately imitate a black-box predictor” and is measured in terms of accuracy score, F1-score, and so on, but wrt synthetic data capturing the behavior of the black-box. Analogously, in Lakkaraju et al. (2019), fidelity concerns the ability of an explanation to “faithfully mimic the behavior” of a model and is assessed in terms of the disagreement between the labels predicted by the explanation and the labels predicted by the model. In the case of explanations concerning a single instance, *local fidelity* has been defined as a measure of how well an explanation model approximates the original model in a neighborhood of the considered instance in need of explaining (Ribeiro et al., 2016; Alvarez-Melis and Jaakkola, 2017). In a similar vein, White and d'Avila Garcez (2020) define *counterfactual fidelity error* as the difference between the actual perturbation of a parameter needed to change the outcome in the original model and an estimate of that value, calculated using an approximate model.

Du et al. (2019) propose a *post-hoc* attribution method to explain the predictions of recurrent neural networks (RNNs) in text mining tasks with the goal of producing explanations, both at word-level and phrase-level which are faithful to the original RNN model. The method is specifically tailored to RNNs' architecture as it resorts to computations on hidden state vectors. Faithfulness is evaluated empirically by computing a score based on the following idea: if one deletes the sentence with the highest attribution for a given prediction, one should then observe a significant drop in the probability of the predicted outcome, if the method is faithful. Thus, this work does not introduce a formal notion of faithfulness which is directly comparable to our characterization of descriptive accuracy and, in fact, the faithfulness score proposed is only indirectly related to the internal behavior of the RNN or of any other classifier.

The work by Adebayo et al. (2018) focuses on saliency methods used to highlight relevant features in images and shows that some of these methods are independent of both the data the model was trained on, and the model parameters, thus pointing out a lack of descriptive accuracy. Interestingly, but not completely surprisingly, it is shown that visual inspection of saliency maps may be misleading and some systematic tests (called sanity checks) are applied to verify whether the explanations depend on the data and the model parameters. The very interesting analysis carried out in this work provides striking evidence that the notion of descriptive accuracy requires more attention, while, differently from our present work, it does not include a proposal for an explicit formalization of this notion.

Yeh et al. (2019) address the problem of defining objective measures to assess explanations and propose, in particular, an infidelity measure, which can be roughly described as the difference between the effect of an input perturbation on the explanation and its effect on the output, and a sensitivity measure capturing the degree to which the explanation is affected by insignificant perturbations. Both measures use the classifier as a black box and hence there are no a priori guarantees about their ability to satisfy descriptive accuracy, as discussed in the present paper. Indeed, the authors apply a sanity check in the spirit of Adebayo et al. (2018) to verify whether the explanations generated to optimize the proposed measures are related to the model.

In the context of deep networks, Sundararajan et al. (2017) propose two axioms called Sensitivity and Implementation Invariance. The former consists of two requirements: (a) for every input and baseline that differ in one feature but have different predictions then the differing feature should be given a non-zero attribution; (b) if the function implemented by the deep network does not depend (mathematically) on some variable, then the attribution to that variable is always zero. The latter states that attributions should be identical for two functionally equivalent networks, where two networks are functionally equivalent if their outputs are equal for all inputs, despite having very different implementations. Sensitivity bears some similarity with the weakest notion we consider, namely naive descriptive accuracy, as they both refer to the role of individual variables and to ensure their relevance when present in explanations. However the perspective is slightly different as we essentially require that the presence of a feature in the explanation is somehow justified by the model, while Sundararajan et al. (2017) require that a feature is present in the explanation under some specific conditions. Bridging these perspectives is an interesting issue for future work. The requirement of Implementation Invariance is motivated by the authors with the claim that attribution can be colloquially defined as assigning the blame (or credit) for the output to the input features. Such a definition does not refer to implementation details. While referring to the special (and rather unlikely in practice) situation where two internally different classifiers produce exactly the same output for the same input, we regard this requirement, which is somehow in contrast with descriptive accuracy, as partly questionable. Indeed, the fact that internal differences are reflected in the explanations may be, at least in principle, useful for some purposes like model debugging. If the differences concern the use of actually irrelevant features, we argue that this aspect should be captured by more general relevance-related criteria.

Chan et al. (2022) carry out a comparative study of faithfulness metrics for model interpretability methods in the context of natural language processing (NLP). Six faithfulness metrics are examined, all of which are based, with different nuances, on an evaluation of the role of the most important tokens in the classified sentences, in particular the common idea underlying these metrics is to compare the output of the classifiers for the same input with or without the most important tokens. These metrics use classifiers as black boxes and do not take into consideration their actual internal operation, so, while sharing the general goal of avoiding explanations that have loose correspondence with the explained model, their scope is somehow orthogonal to ours. Chan et al. (2022) observe that, though referring to the same basic principle, these metrics may provide contradictory outcomes, so that the most faithful method according to a metric is the worst with respect to another one. To address this problem, the authors propose a property of Diagnosticity, which refers to the capability of a metric to discriminate a more faithful interpretation from an unfaithful one (where, in practice, randomly generated interpretations are used as instances of unfaithful ones). Applying a possibly adapted notion of Diagnosticity in the context of our proposal appears an interesting direction of future work.

Mollas et al. (2022) propose *Altruist*, an approach for transforming the output of feature attribution methods into explanations using argumentation based on classical logic. In particular, *Altruist* is able to distinguish truthful vs. untruthful parts in a feature attribution and can work as a meta-explanation technique on top of a set of feature attribution methods. Similarly to our proposal, Mollas et al. (2022) assume that the importance weights produced by feature attribution methods are typically associated with a monotonic notion, and that end-users expect monotonic behavior when altering the value of some feature. On this basis, *Altruist* includes a module which assesses the truthfulness of an importance value by comparing the expected changes of the output, given some perturbations, with respect to the actual ones and then building an argumentation framework which is based on the predicates corresponding to the results of these comparisons and can be used to support a dialogue with the final user. The notion of truthfulness used in Mollas et al. (2022) refers to correspondence with users expectations rather than with internal model behavior and is thus complementary to our notion of descriptive accuracy. As both aspects are important in practice, bridging them and investigating their relationships is a very interesting direction of future work. Also, the uses of argumentation in the two works are somehow complementary: while we resort to argumentation concepts as foundational notions, in Mollas et al. (2022) logic-based argumentation frameworks are used to support reasoning and dialogues about truthfulness evaluations.

Overall, whereas formal counterparts of predictive accuracy/faithfulness/fidelity have been extensively studied in the XAI literature, to the best of our knowledge, formal counterparts of DA appear to have received limited attention up to now. This gap is particularly significant for the classes of *post-hoc* explanations methods which, *per se*, have no relations with the underlying operation of the explained model and therefore cannot rely on any implicit assumption that DA is guaranteed, in a sense, by construction. This applies, in particular, to the family of *model-agnostic local explanation* methods, namely methods which are designed to be applicable to any model (and hence need to treat the model itself purely as a black-box) and whose explanations are restricted to illustrate individually a single outcome of the model without aiming to describe its behavior in more general terms. This family includes the well-known class of *additive feature attribution* methods, such as LIME (Ribeiro et al., 2016) and SHAP (Lundberg and Lee, 2017), where the explanation for the outcome of a model basically consists in ascribing to each input feature a numerical weight. We will study our formalisations of DA in the context of both LIME and SHAP.

SHAP has been shown to be the only additive attribution method able to jointly satisfy three formal properties, called *local accuracy, missingness*, and *consistency* (see Lundberg and Lee, 2017 for details). These properties do not directly concern the internal working of the model and thus cannot be seen as forms of DA. Indeed, our analysis will show that SHAP, as well as LIME, are not guaranteed to satisfy our notions of DA– thus local accuracy, missingness and consistency do not suffice to enforce DA in our sense.

A variety of approaches devoted in particular to the explanation of Bayesian networks exist in the literature (Lacave and Díez, 2002; Mihaljevic et al., 2021). At a high level these approaches can be partitioned into three main families (Lacave and Díez, 2002): explanation of evidence (which concerns explaining observations by abducing the value of some unobserved variables), explanation of model (which aims at presenting the entire underlying model to the user), and explanation of reasoning. Explanation of reasoning is the one that best lends itself to fulfilling DA. According to Lacave and Díez (2002), it is in turn divided into: (i) explanation of the results obtained by the system and the reasoning process that produced them; (ii) explanation of the results not obtained by the system, despite the user's expectations; (iii) hypothetical reasoning, i.e., what results the system would have returned if one or more given variables had taken on different values from those observed. Our DARX approach is mainly related to point (i), even if it may support some form of hypothetical reasoning too. We remark that the spirit of DARX is not advancing the state of the art in explanations for Bayesian networks but rather providing a concrete example of a method satisfying the DA properties we introduce and showing that even with this baseline approach we can get improvements with respect to popular model-agnostic methods, as concerns satisfaction of DA.

To introduce formal notions of DA we take inspiration from basic concepts in formal argumentation. As pointed out by Cyras et al. (2021), many popular methods for generating explanations in AI can be seen as implicitly argumentative, in addition to the vast literature on overtly argumentative approaches. These include the use of a variety of argumentation frameworks for explanation purposes, as surveyed in particular by Cyras et al. (2021), with a broad set of application domains, ranging from law and medical informatics to robotics and security, as discussed by Vassiliades et al. (2021). As pointed out by Cyras et al. (2021), however, in the literature the study of formal properties of argumentation frameworks have received by far more attention than the investigation of desirable properties of explanations, and our use of argumentation to drive the definition of explanation requirements, rather than of the explanation methods themselves, appears to be a novel investigation line to the best of our knowledge.

Last but not least, the human-centric approach requires that users' perspectives lie at the heart of the evaluation of AI explanation methods. Some works have identified properties naturally amenable to being assessed with humans, for example, Murdoch et al. (2019) propose *relevancy*, concerning the ability to provide insight for a particular audience into a chosen domain problem. It is widely acknowledged though that more user testing would be beneficial for evaluating XAI methods (e.g., see Keane et al., 2021). We contribute to this line of research by conducting a user study to assess whether our dialectical DA is in line with user expectations.

## 4. Preliminaries on probabilistic classifiers

As DA is inherently related to the internal operation of a model, rather than just to its input/output behavior, any formal notion of DA cannot be completely model-agnostic. It follows that an investigation of DA needs to find a balance between the obvious need of wide applicability and the potential advantages of model-tailored definitions. For this reason we will focus on the broad family of *probabilistic classifiers*.

We consider (discrete) probabilistic classifiers with *feature variables* **X** = {*X*_1_, …, *X*_*m*_} (*m*>1) and *class variables* **C** = {*C*_1_, …, *C*_*n*_} (*n*≥1). Each (random) variable *V*_*i*_∈**V** = **X**∪**C** is equipped with a discrete set of possible *values*^2^ Ω_*V*_*i*__: we define the *feature space* as $X=\Omega_{X_{1}}$ × … × Ω_*X*_*m*__ and the *class space* as $C=\Omega_{C_{1}}\times \ldots \times \Omega_{C_{n}}$. From now on, we call any vector **x** ∈ X an *input* and denote as **x**(*X*_*i*_) the value of feature *X*_*i*_ in **x**. Given input **x**, a *probabilistic classifier* PC computes, for each class variable *C*_*i*_ and value ω∈Ω_*C*_*i*__ , the probability *P*(*C*_*i*_ = ω|**x**) that *C*_*i*_ takes value ω, given **x**.^3^ We then refer to the *resulting value* for a class variable *C*_*i*_∈**C** given input **x** as $\mathrm{PC}\left(C_{i}|\text{x}\right)=argmax_{\omega \in \Omega_{C_{i}}}P\left(C_{i}=\omega |\text{x}\right)$. Table 1 gives a probabilistic classifier for a (toy) financial setting where the values of class variables *problematic external* e*vent* and *drop in* c*onsumer confidence* are determined based on the feature variables *company* s*hare price trend*, d*evaluation of currency, healthy* h*ousing market* and *negative breaking* n*ews cycle*. Here, for any variable *V*_*i*_∈**V**, Ω_*V*_*i*__ = {+, −}.

**Table 1:** An example of *probabilistic classifier* with **X** = {*s, d, h, n*} and **C** = {*c, e*}.

*s*	+	+	+	+	+	+	+	+	−	−	−	−	−	−	−	−
*d*	+	+	+	+	−	−	−	−	+	+	+	+	−	−	−	−
*h*	+	+	−	−	+	+	−	−	+	+	−	−	+	+	−	−
*n*	+	−	+	−	+	−	+	−	+	−	+	−	+	−	+	−
*c*	+	−	+	+	+	−	+	−	+	−	+	+	+	−	+	−
*P*	.60	.65	1	.60	.60	1	1	.65	.60	.65	1	.60	.60	1	1	.65
*e*	+	−	+	+	+	−	+	−	+	−	+	+	+	−	+	−
*P*	.60	1	.60	.60	.60	1	.60	1	1	.65	1	1	1	.65	1	.65

Here, e.g., for x (highlighted in bold) such that x(*s*) = x(*d*) = x(*h*) = x(*n*) = +, PC(c|x)=+ (as *P*(*c* = +|x) =.60), and PC(e|x)=+ (as *P*(*e* = +|x) =.60).

For *X*_*i*_∈**X**, we will abuse notation as follows, to simplify some of the formal definitions later in the paper: $\mathrm{PC}\left(X_{i}|\text{x}\right)=\text{x}\left(X_{i}\right)$ (basically, the “resulting value” for a feature variable, given an input, is the value assigned to that variable in the input) and *P*(*X*_*i*_ = **x**(*X*_*i*_)) = 1 (basically, the probability of a feature variable being assigned its value, in the given input, is 1). We will also use notation:


$$P(V=v|x,\;set(V_{i}=v_{i}))=\;\{\begin{array}{ll} P(V=v|x^{\prime }), & {\text{if }V}_{i}\in \text{ X, }\\ P(V=v|x,{\;V}_{i}=v_{i}), & {\text{if }V}_{i}\in \text{ C}, \end{array}$$

where, in the first case, ${\text{x}}^{\prime }\left(V_{i}\right)=v_{i}$ and ${\text{x}}^{\prime }\left(V_{j}\right)=\text{x}\left(V_{j}\right)$ for all *V*_*j*_∈**X**\{*V*_*i*_}. Basically, this notation allows to gauge the effects of changes in value for (input or class) variables on the probabilities computed by the classifiers (for assignments of values to any variables).

Various types of probabilistic classifiers exist. In Section 7 we will experiment with (explanations for) a variety of (discrete) *Bayesian Classifiers* (BCs, see Bielza and Larrañaga, 2014 for an overview), where the variables in **V** constitute the nodes in a Bayesian network, i.e., a directed acyclic graph whose edges indicate probabilistic dependencies amongst the variables.^4^ We will also experiment with (explanations for) *chained probabilistic classifiers* (CCs, e.g., as defined by Read et al. (2009) for the case of BCs). These CCs result from the combination of simpler probabilistic classifiers (possibly, but not necessarily, BCs), using an ordering ≻_*C*_ over **C** such that the value of any *C*_*i*_∈**C** is treated as a feature value for determining the value of any *C*_*j*_∈**C** with *C*_*j*_≻_*C*_*C*_*i*_, and thus a classifier computing values for *C*_*i*_ can be chained with one for computing values for *C*_*j*_. For illustration, in Table 2 we re-interpret the classifier from Table 1 as a CC amounting to a chain of two classifiers, using *e*≻_*C*_*c*: the classifier (a) determines the value of *c* as an additional input for the classifier (b). Then, the overall classifier determines the value of *c* first based on the feature variables *d*, *h* and *n*, and then *e* based on *s* and *c* (treated as a feature variable in the chaining, thus implicitly taking into account *d*, *h* and *n*). Note that, in Table 2 and throughout the paper, we abuse notation and use inputs for overall (chained) classifiers (**x** in the caption of the table) as inputs of all simpler classifiers forming them (rather than the inputs' restriction to the specific input variables of the simpler classifiers).

**Table 2:** An example of *chained probabilistic classifier* (CC) with **(a)** the first probabilistic classifier ${\mathrm{PC}}_{1}$ with **X**_1_ = {*d, h, n*}, **C**_1_ = {*c*}, and **(b)** the second probabilistic classifier ${\mathrm{PC}}_{2}$ with **X**_2_ = {*s, c*}, **C**_2_ = {*e*} (both inputs highlighted in bold).

(a)								
*d*	**+**	+	+	+	−	−	−	−
*h*	**+**	+	−	−	+	+	−	−
*n*	**+**	−	+	−	+	−	+	−
*c*	+	−	+	+	+	−	+	−
*P*	.60	.65	1	.60	.60	1	1	.65
**(b)**								
*s*	**+**		+		−		−	
*c*	**+**		−		+		−	
*e*	+		−		+		−	
*P*	.60		1		1		.65	
**(c)**								
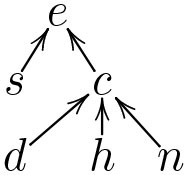								

Here, e.g., for **x** as in the caption of Table 1, $\mathrm{PC}\left(c|\text{x}\right)={\mathrm{PC}}_{1}\left(c|\text{x}\right)=+$ and $\mathrm{PC}\left(e|\text{x}\right)={\mathrm{PC}}_{2}\left(e|\text{x},set\left(c={\mathrm{PC}}_{1}\left(c|\text{x}\right)\right)\right)=+$. (c) A structural description for the CC in **(a, b)**, shown as a graph.

For some families of probabilistic classifiers (e.g., for BCs) it is possible to provide a graphical representation which gives a synthetic view of the dependence and independence relations between the variables. In these cases, we will assume that the classifier is accompanied by a *structural description*, namely a set SD⊆V×V. The structural description identifies for each variable *V*_*j*_∈**V** a (possibly empty) set of *parents* $\mathrm{PA}\left(V_{j}\right)=\left\{V_{i}|\left(V_{i},V_{j}\right)\right\}\in \mathrm{SD}$ with the meaning that the evaluation of *V*_*j*_ is completely determined by the evaluations of $\mathrm{PA}\left(V_{j}\right)$ in the classifier. In the case of BCs, the parents of each (class) variable correspond to the variables in its unique *Markov boundary* (Pearl, 1989; Neapolitan and Jiang, 2010) $M:\text{V}\to 2^{\text{V}}$, where, for any *V*_*i*_∈**V**, $M\left(V_{i}\right)$ is the ⊆-minimal set of variables such that *V*_*i*_ is conditionally independent of all the other variables ($\text{V}\backslash M\left(V_{i}\right)$), given $M\left(V_{i}\right)$. In the case of CCs, even when no information is available about the internal structure of the individual classifiers being chained, a structural description may be extracted to reflect the connections between features and classes. For illustration, for the CC in Tables 2a, b, the structural description is SD={(d,c),(h,c),(n,c),(s,e),(c,e)}, given in Table 2c as a graph.

We remark that notions similar to structural descriptions have been considered earlier in the literature. For instance, in Timmer et al. (2015) the argumentative notion of a support graph derived from a Bayesian network has been considered. This support graph however is built with reference to a given variable of interest and is meant to facilitate the construction of arguments which provide a sort of representation of the reasoning inside the network. In our case we provide a structural description which does not refer to a single variable of interest and is not used for building explanations but rather to verify whether they satisfy structural DA, as will be described later.

In the remainder, unless specified otherwise, we assume as given a probabilistic classifier PC with feature variables **X** and class variables **C**, without making any assumptions.

## 5. Formalizing descriptive accuracy

We aim to define DA, using argumentative notions as a basis, in a way which is independent of any specific explanation method (but with a focus on the broad class of local explanations, and specifically feature attribution methods to obtain them).

At a very abstract level, an explanation, whatever its structure is, can be regarded as including a set of *explanation elements* which are provided by the explainer to the explainee in order to justify some system *outcome*. Relationships between explanations under this abstract understanding and argumentative notions can be drawn at different levels. According to a first basic interpretation, the main components of an explanation can be put in correspondence with the essential parts of *Toulmin's argument model* (Toulmin, 1958): the system outcome can be regarded as an argument *claim*, while the explanation elements are the *data* supporting the claim; claim and data are connected (implicitly) by a *warrant*, namely the assumption on which the validity of the link from the data to the claim relies. In a more articulated interpretation, one can consider the existence of distinct argumentative relations underlying the explanation. Specifically, as mentioned in Section 2, we will focus on the fundamental relations of *attack* and *support* encompassed in *bipolar argumentation frameworks* (Amgoud et al., 2008).

According to both interpretations, the property of DA can be understood as the requirement that the argumentative structure underlying the explanation has a correspondence in the system being explained, and hence can be regarded as accurate. In particular, in the basic interpretation, we regard an explanation as satisfying DA if a suitable warrant, linking the explanation elements with the outcome, can be identified in the behavior of the system, while in the more articulated interpretation we require that the relations of attack and support correspond to the existence of suitable bipolar influences within the system.

In order to convert these high-level considerations into formal definitions for both argumentative interpretations, we will consider different abstractions of the notion of (local) explanation, able to encompass a broad range of existing notions in the literature as instances. The abstractions we define are based on the combinations of alternative choices along two dimensions. On one hand, we consider two basic elements that an explanation may refer to: (1) *input features*; (2) pairs of variables representing *relations* between variables. When only input features are used then the resulting explanations are flat/shallow, only describing input/output behavior, whereas the inclusion of relations potentially allows for deeper explanation structures. On the other hand, we assume that the basic elements inside an explanation can be: (a) regarded as an undifferentiated set (we call these elements *unsigned*, in contrast with (b)); (b) partitioned into two sets according to their *positive or negative* role in the explanation. The combinations (1)-(a) and (2)-(a) will correspond respectively to the abstract notions of *unipolar* and *relational unipolar* explanations while the combinations (1)-(b) and (2)-(b) will correspond respectively to the notions of *bipolar* and *relational bipolar* explanations.^5^

Driven by argumentative interpretations for these forms of explanations, in terms of Toulmin's argument model and bipolar argumentation as highlighted above, we will introduce a notion of *naive* DA for all the kinds of abstract explanations we consider and a notion of *dialectical* DA tailored to the two cases of relational explanations. We see naive DA as a very weak pre-requisite for explanations, and prove that it is implied by dialectical DA for both bipolar and relational bipolar explanations (Propositions 1 and 2, resp.): thus, naive DA can be seen as a step toward defining dialectical DA. (Naive and) Dialectical DA are applicable to *any* probabilistic classifiers. In the specific setting of classifiers with underlying graph structures, such as BCs and CCs, we will also define a notion of *structural* DA for relational unipolar/bipolar explanations. Table 3 summarizes the definitions from this section, given below.

**Table 3:** Explanations with the characteristics they hold (as combinations of (1)-(2) and (a)-(b)) represented by ✓ and their DA properties (in italics) represented by ⋆.

	**Unip**.	**Rel. Unip**.	**Bip**.	**Rel. Bip**.
	**(Section 5.1)**	**(Section 5.2)**	**(Section 5.3)**	**(Section 5.4)**
(1) input features	✓		✓	
(2) relations		✓		✓
(a) unsigned	✓	✓		
(b) positive or negative			✓	✓
*Basic DA*	⋆	⋆	⋆	⋆
*Dialectical DA*			⋆	⋆
*Structural DA*		⋆		⋆

### 5.1. Unipolar explanations and naive DA

We begin with a very general notion of *unipolar explanation*: we only assume that, whatever the nature and structure of the explanation, it can be regarded at an abstract level as a *set of features*:

** Definition 1**. Given an input x∈X and the resulting value ω=PC(C|x) for class *C*∈**C** given **x**, a *unipolar explanation* (for *C* = ω, given **x**) is a triple 〈**F**, *C*, **x**〉 where **F**⊆**X**.

It is easy to see that it is straightforward to derive unipolar explanations from the outcomes produced by existing explanation methods when they return features accompanied by additional information (e.g., feature importance as in the case of the attribution methods LIME and SHAP): basically, in these settings the unipolar explanations disregard the additional information, and amount to (a subset of) the set of features alone (e.g., the *k* most important features).

From an argumentative perspective, the features in a unipolar explanation can be regarded as the grounds (somewhat in Toulmin's sense) for justifying the resulting value assigned by the classifier to a class variable, for the input under consideration. Accordingly, we require that some form of warrant justifying the link of these grounds with the resulting value can be identified. This corresponds to the simplest form of DA, i.e., *naive DA*, whose intuition is that the features included in a unipolar explanation should be “relevant,” i.e., should play a role in the underlying model, as formally defined in the following. Property 1.

A unipolar explanation 〈**F**, *C*, **x**〉 satisfies *naive descriptive accuracy* iff for every *X*_*i*_∈**F** there exists an input ${\text{x}}^{\prime }\in X$ with ${\text{x}}^{\prime }\left(X_{j}\right)=\text{x}\left(X_{j}\right)$ for every *X*_*j*_≠*X*_*i*_ and with ${\text{x}}^{\prime }\left(X_{i}\right)\neq \text{x}\left(X_{i}\right)$, such that, letting ω=PC(C|x), it holds that *P*(*C* = ω|**x**)≠*P*(*C* = ω|**x**′).

Naive DA holds when, for each individual feature, there is at least one case (i.e., an alternative input **x**′ to the input **x** being explained) where a change in the value of the feature has an effect on the probability of the value of the class variable: thus, it is a rather weak requirement as it excludes individually “irrelevant” features from playing a role in the explanation. Note that this property can also be interpreted as a rudimentary form of *counterfactual reasoning* (of the form “what happens when the value of some variable changes?”). However, it is too weak to define counterfactual explanations (e.g., as first modeled in Tolomei et al., 2017; Wachter et al., 2017). Indeed, changes in probabilities, as in naive DA, may not lead to changes in classification, as required when defining counterfactual explanations. Furthermore, the notion of naive DA disregards considerations of “actionability” for counterfactual explanations, e.g., as addressed by Karimi et al. (2021). We leave formalization of DA for counterfactual explanations to future work.

For illustration, given the probabilistic classifier in Table 1 and **x** as in the table's caption, the unipolar explanation 〈{*s, d, h, n*}, *c*, **x**〉 does not satisfy naive DA, given that both *s* and *d* are “irrelevant” here: changing the value of either does not affect the probability of *c*. Instead, it is easy to see that 〈{*h, n*}, *c*, **x**〉 satisfies naive DA.

### 5.2. Bipolar explanations and dialectical DA

Unipolar explanations consist of “minimal” information, i.e., just the features playing a role in explanations. At a finer level of granularity, corresponding to a greater degree of articulated argumentative interpretation, we consider *bipolar explanations*, where the features are partitioned into two sets: those having a positive, or *supporting*, effect on the resulting value and those having a negative, or *attacking*, effect. The notions of positive and negative effect may admit different specific interpretations in different contexts, the general underlying intuition being that the corresponding features provide, resp., reasons for and against the resulting value being explained. Whatever the interpretation, we assume that positive and negative features are disjoint, as a feature with a twofold role in an explanation could be confusing for the user.

** Definition 2**. Given an input x∈X and the resulting value ω=PC(C|x) for class *C*∈**C** given **x**, a *bipolar explanation* (for *C* = ω, given **x**) is a quadruple 〈**F**_+_, **F**_−_, *C*, **x**〉 where **F**_+_⊆**X**, **F**_−_⊆**X**, and **F**_+_∩**F**_−_ = ∅ ; we refer to features in **F**_+_ and **F**_−_ resp. as *positive* and *negative reasons*.

It is easy to see that existing explanation methods can be regarded as producing bipolar explanations when those methods return features accompanied by additional positive or negative information (e.g., positive and negative feature importance as in the case of attribution methods such as LIME and SHAP): in these settings, as in the case of unipolar explanations, bipolar explanations disregard the additional information, and amount to (a subset of) the set of features with their polarity (e.g., the *k* features with the highest positive importance as positive features and the *k* features with the lowest negative importance as negative features).

Taking into account the distinction between positive and negative reasons, we introduce a property requiring that the dialectical role assigned to features is justified: Property 2. A bipolar explanation 〈**F**_+_, **F**_−_, *C*, **x**〉 satisfies *dialectical descriptive accuracy* iff for every *X*_*i*_∈**F**_+_∪**F**_−_, for every ${\text{x}}^{\prime }\in X$ with ${\text{x}}^{\prime }\left(X_{j}\right)=\text{x}\left(X_{j}\right)$ for all *X*_*j*_≠*X*_*i*_ and ${\text{x}}^{\prime }\left(X_{i}\right)\neq \text{x}\left(X_{i}\right)$, letting ω=PC(C|x), it holds that if *X*_*i*_∈**F**_+_ then *P*(*C* = ω|**x**)>*P*(*C* = ω|**x**′); if *X*_*i*_∈**F**_−_ then *P*(*C* = ω|**x**) < *P*(*C* = ω|**x**′).

In words, if a feature is identified as a positive (negative) reason for the resulting value for a class variable, given the input, the feature variable's value leads to increasing (decreasing, resp.) the posterior probability of the class variable's resulting value (with all other feature values unchanged). This has a direct correspondence with the properties of monotonicity considered in the literature for gradual argumentation semantics (Amgoud and Ben-Naim, 2018; Baroni et al., 2019) and we posit that this requirement ensures that each reason has a cognitively plausible dialectical meaning, faithful to human intuition, as we will examine in Section 8.

For illustration, in the running example with PC in Table 1, the bipolar explanation 〈{*d, n*}, {*h*}, *c*, **x**〉, given input **x** as in the table's caption does not satisfy dialectical DA. Indeed, *d* is a positive reason in the explanation but, for **x**′ agreeing with **x** on all features other than *d* (with **x**′(*d*) = −), we obtain *P*(*c* = +|**x**) =.60≮*P*(*c* = +|**x**′) =.60. Instead, it is easy to see that the bipolar explanation 〈{*n*}, {*h*}, *c*, **x**〉, satisfies dialectical DA.

Note that the property of dialectical DA may not be satisfied by all re-interpretations of existing forms of explanations as bipolar explanations. As an example, consider *contrastive explanations* of the form proposed by Dhurandhar et al. (2018). Here, features are split into *pertinent positives and negatives*, which are those whose presence or absence, resp., is “relevant” to the resulting value being explained. If these pertinent positives and negatives are understood, resp., as positive and negative reasons in bipolar explanations, the latter do not satisfy dialectical DA, since both positive and negative pertinent features support the resulting value being explained. If, instead, pertinent positives and negatives are both understood as positive reasons, then the resulting bipolar explanations may satisfy dialectical DA: we leave the analysis of this aspect, and the definition of additional forms of DA e.g., able to distinguish between pertinent positives and negatives, for future work.

In general, unipolar explanations can be directly obtained from bipolar explanations by ignoring the distinction between positive and negative reasons, and the property of naive DA can be lifted:

** Definition 3**. A *bipolar explanation* 〈**F**_+_, **F**_−_, *C*, **x**〉 *satisfies naive descriptive accuracy* iff the unipolar explanation 〈**F**_+_∪**F**_−_, *C*, **x**〉 satisfies naive descriptive accuracy.

It is then easy to see that dialectical DA strengthens naive DA:^6^

** Proposition 1**. If a bipolar explanation 〈**F**_+_, **F**_−_, *C*, **x**〉 satisfies dialectical DA then it satisfies naive DA.

### 5.3. Relational unipolar explanations and naive DA

Moving toward a notion of deeper explanations, we pursue the idea of providing a more detailed view of the relations between variables of a probabilistic classifier, reflecting influences possibly occurring amongst them. To this purpose, we first introduce *relational unipolar explanations* as follows.

** Definition 4**. Given x∈X and the resulting value ω=PC(C|x) for *C*∈**C** given **x**, a *relational unipolar explanation* (for *C* = ω, given **x**) is a triple 〈R,C,x〉 where R⊆V×V.

In words, a relational unipolar explanation includes a set R of pairs of variables (i.e., a relation between variables) where $\left(V_{i},V_{j}\right)\in R$ indicates that the value of *V*_*i*_ has a role in determining the value of *V*_*j*_, given the input.

For illustration, for PC in Table 1 , 〈{(*s, e*), (*c, e*)}, *e*, **x**〉 may be a relational unipolar explanation for **x** in the table's caption, indicating that *s* and *c* both influence (the value of) *e*. Note that relational unipolar explanations admit unipolar explanations as special instances: given a unipolar explanation 〈**F**, *C*, **x**〉, it is straightforward to see that 〈**F**×{*C*}, *C*, **x**〉 is a relational unipolar explanation. However, as demonstrated in the illustration, relational unipolar explanations may include relations besides those between feature and class variables found in unipolar explanations. From an argumentative perspective, this corresponds to regarding the explanation as composed by a set of “finer grain” arguments, identifying not only the grounds for the explained outcome, but also for intermediate evaluations of the classifier, which in turn may provide grounds for the explained outcome and/or other intermediate evaluations.

The notion of naive DA can be naturally extended to relational unipolar explanations by requiring that a warrant based on relevance can be identified for each of the (implicit) finer arguments. Property 3. A relational unipolar explanation 〈R,C,x〉 satisfies *naive descriptive accuracy* iff for every $\left(V_{i},V_{j}\right)\in R$, letting $v_{i}=\mathrm{PC}\left(V_{i}|\text{x}\right)$ and $v_{j}=\mathrm{PC}\left(V_{j}|\text{x}\right)$, there exists $v_{i}^{\prime }\in \Omega_{V_{i}}$, $v_{i}^{\prime }\neq v_{i}$, such that $P\left(V_{j}=v_{j}|\text{x}\right)\neq P\left(V_{j}=v_{j}|\text{x},set\left(V_{i}=v_{i}^{\prime }\right)\right)$.

For illustration, for PC in Table 1, 〈{(*s, e*), (*n, e*)}, *e*, **x**〉 satisfies naive DA for **x** in the table's caption, but 〈{(*s, e*), (*d, e*)}, *e*, **x**〉 does not, as changing the value of *d* to − (the only alternative value to +), the probability of *e* = + remains unchanged.

It is easy to see that, for relational unipolar explanations 〈**F**×{*C*}, *C*, **x**〉, corresponding to unipolar explanations 〈**F**, *C*, **x**〉, Property 1 is implied by Property 3.

### 5.4. Relational bipolar explanations and dialectical DA

Bipolarity can be directly enforced on relational explanations as follows.

** Definition 5**. Given an input x∈X and the resulting value ω=PC(C|x) for class *C*∈**C** given **x**, a *relational bipolar explanation (RX)* is a quadruple $\langle R_{+},R_{-},C,\text{x}\rangle$ where: $R_{+}\subseteq \text{V}\times \text{V}$, referred to as the set of *positive reasons*; $R_{-}\subseteq \text{V}\times \text{V}$, referred to as the set of *negative reasons*; $R_{+}\cap R_{-}=\emptyset$.

An RX can be seen as a graph of variables connected by edges identifying positive or negative reasons, i.e., as a bipolar argumentation framework (Cayrol and Lagasquie-Schiex, 2005). Here DA consists in requiring that the polarity of each edge is justified, which leads to the following definition, extending to relations the idea expressed in Property 2. Property 4. An RX $\langle R_{+},R_{-},C,\text{x}\rangle$ satisfies *dialectical descriptive accuracy* iff for every $\left(V_{i},V_{j}\right)\in R_{+}\cup R_{-}$, letting $v_{i}=\mathrm{PC}\left(V_{i}|\text{x}\right)$, $v_{j}=\mathrm{PC}\left(V_{j}|\text{x}\right)$, it holds that, for every $v_{i}^{\prime }\in \Omega_{V_{i}}\backslash \left\{v_{i}\right\}$:

if $\left(V_{i},V_{j}\right)\in R_{+}$ then $P\left(V_{j}=v_{j}|\text{x}\right)>P\left(V_{j}=v_{j}|\text{x},set\left(V_{i}=v_{i}^{\prime }\right)\right)$;

if $\left(V_{i},V_{j}\right)\in R_{-}$ then $P\left(V_{j}=v_{j}|\text{x}\right)<P\left(V_{j}=v_{j}|\text{x},set\left(V_{i}=v_{i}^{\prime }\right)\right)$.

Similarly to dialectical descriptive accuracy for bipolar explanations, if, given the input, a variable *V*_*i*_ is categorized as a positive (negative) reason for the resulting value of another variable *V*_*j*_, *V*_*i*_'s value leads to increasing (decreasing, resp.) the posterior probability of *V*_*j*_'s resulting value (with all values of the other variables playing a role in *V*_*j*_'s value remaining unchanged).

**Figure 1:**
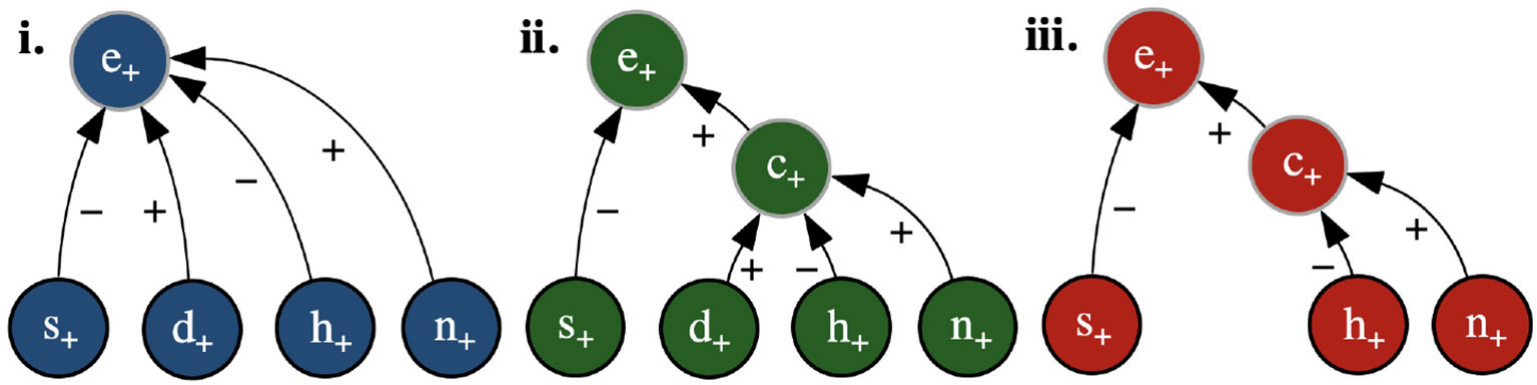
Example RXs (shown as graphs, with positive and negative reasons given by edges labeled + and −, resp.) with input **x** such that **x**(*s*) = **x**(*d*) = **x**(*h*) = **x**(*n*) = + (represented as *s*_+_, *d*_+_, *h*_+_, *n*_+_) and (resulting) class values *c* = + (represented as *c*_+_) and *e* = + (represented as *e*_+_).

Examples of RXs for the running example are shown as graphs in Figure 1 (where the nodes also indicate the values ascribed to the feature variables in the input **x** and to the class variables by any of the toy classifiers in Tables 1, 2). Here, (iii) satisfies dialectical DA, since setting to − the value of any variable with a positive (negative) reason to another variable will reduce (increase, resp.) the probability of the latter's value being +, whereas (ii) does not, since setting *d* to − does not affect the probability of *c*'s value being + and (i) does not since setting *d* to − does not affect the probability of *e*'s value being +.

Similarly to the case of unipolar/bipolar explanations, relational unipolar explanations can be directly obtained from RXs by ignoring the distinction between positive and negative reasons, and the property of dialectical DA can be lifted:

** Definition 6**. An *RX* $\langle R_{+},R_{-},C,\text{x}\rangle$ *satisfies naive descriptive accuracy* iff the relational unipolar explanation $\langle R_{+}\cup R_{-},C,\text{x}\rangle$ satisfies naive descriptive accuracy.

It is then easy to see that dialectical DA strengthens naive DA:

** Proposition 2**. If an RX $\langle R_{+},R_{-},C,\text{x}\rangle$ satisfies *dialectical DA* then it satisfies naive DA.

Note that bipolar explanations 〈**F**_+_, **F**_−_, *C*, **x**〉 can be regarded as special cases of RXs, i.e., 〈{(*X, C*)∣*X*∈**F**_+_}, {(*X, C*)∣*X*∈**F**_−_}, *C*, **x**〉 (indeed, the RX in Figure 1i is a bipolar explanation). Thus, from now on we will often refer to all forms of bipolar explanation as RXs.

### 5.5. Relational explanations and structural DA

When a classifier is equipped with a structural description, one can require that the relations used for explanation purposes in RXs are subsets of those specified by the structural description, so that the RXs correspond directly to (parts of) the inner working of the model. This leads to the following additional form of DA: Property 5. Given a probabilistic classifier PC with structural description SD:

- a relational unipolar explanation 〈R,C,x〉 satisfies *structural descriptive accuracy* iff R⊆SD; and
- an RX $\langle R_{+},R_{-},C,\text{x}\rangle$ satisfies *structural descriptive accuracy* iff $R_{+}\cup R_{-}\subseteq \mathrm{SD}$.

For instance, suppose that SD is the structural description in Table 2c. Then, the RXs in Figures 1ii, iii satisfy structural DA, since all of the relations are contained within the structural description, while the RX in Figure 1i does not, since the relations from *d*, *h* and *n* to *e* are not present in the structural description.

## 6. Achieving descriptive accuracy in practice

In this section, we study the satisfaction of the proposed properties by explanation methods. We focus in particular on two existing methods in the literature, namely LIME (Ribeiro et al., 2016) and SHAP (Lundberg and Lee, 2017) , and variants thereof that we design so that they satisfy structural DA. After showing that none of these methods satisfies all the properties introduced in Section 5, we introduce a novel form of explanation guaranteed to satisfy them, by definition. Thus, this novel form of explanation can be seen as a “champion” for our proposed forms of DA, showing that they can be satisfied in practice.

We start with LIME and SHAP. The explanations they produce (given an input **x** and a classifier, computing *C* = ω, given **x**) basically consist in computing, for each feature *X*_*i*_∈**X**, a real number *w*(**x**, *X*_*i*_, *C*) indicating the importance of *X*_*i*_, which has assigned value **x**(*X*_*i*_) in the given input **x**, toward the probability of the class variable *C* being assigned value ω=PC(C|x) by the classifier, in the context of **x**.^7^ The absolute value of this number can be interpreted as a measure of the feature importance in the explanation, while its sign, in the context of explaining probabilistic classifiers, indicates whether the feature has a positive or negative role wrt the classifier's resulting value for the explained instance. Features which are assigned a value of zero can be regarded as irrelevant.^8^ Clearly, such explanations correspond to bipolar explanations 〈**F**_+_, **F**_−_, *C*, **x**〉 as in Definition 2, with

- **F**_+_ = {*X*_*i*_∈**X**∣*w*(**x**, *X*_*i*_, *C*)>0} and
- **F**_−_ = {*X*_*i*_∈**X**∣*w*(**x**, *X*_*i*_, *C*) < 0}.

In the remainder, with an abuse of terminology, we call these bipolar explanations *LIME/SHAP explanations*, depending on whether *w* is calculated using, resp., the method of LIME/SHAP. For illustration, consider the classifier in Table 1 and **x** such that **x**(*s*) = **x**(*d*) = **x**(*h*) = **x**(*n*) = +, as in the caption of Figure 1, for which the classifier computes *e* = +. In this simple setting, SHAP computes *w*(**x**, *s*_+_, *e*_+_) = −0.20, *w*(**x**, *d*_+_, *e*_+_) = 0.03, *w*(**x**, *h*_+_, *e*_+_) = −0.05, and *w*(**x**, *n*_+_, *e*_+_) = 0.25 (adopting here the same conventions on variable assignments as in the caption of the Figure). This results in the SHAP explanation in Figure 1i. Thus features *d* and *n* (with their current values) are ascribed positive roles and *s* and *h* are ascribed negative roles in determining the outcome PC(e|x)=+. However, as stated earlier, for feature *d* this is in contrast with the property of naive DA. In fact, by inspection of Table 1, it can be noted that changing the value of this variable individually we would still have *P*(*e* = +|**x**) = 1. To put it in intuitive terms, assigning a positive importance to this variable suggests to the user that its current value (namely +) has a role (though minor) in determining the outcome *e* = +, which is misleading. The following proposition generalizes these considerations:

** Proposition 3**. In general, LIME and SHAP explanations are not guaranteed to satisfy naive nor dialectical DA.

The illustration above proves this result for SHAP explanations, by providing a counterexample to naive (and hence dialectical) DA in the context of the classifier in Table 1. The result for LIME explanations can be proved by introducing spurious features within trained probabilistic classifiers and showing that they play a role within LIME (see Appendix 1). As a side observation, in the appendix we also show empirically that approximate implementations of SHAP (the ones being used in practice, as an exact implementation of SHAP is practically unfeasible) also violate naive (and hence dialectical) DA.

Concerning structural DA, LIME and SHAP explanations may in general satisfy it only if X×C⊆SD, i.e., if the structural description includes all the possible relations from feature variables to class variables. This is, for instance, the case for naive BCs (Maron and Kuhns, 1960), but not for more general BCs or CCs. To overcome this limitation, generalizations of LIME and SHAP explanations can be defined so that they are guaranteed to satisfy structural DA by construction. This requires that the computation of (LIME/SHAP) *w* is applied not only to pairs with a feature and a class, but also, more generally, to any pairs of variables, following the underpinning structural description: in this way a bipolar argumentation framework satisfying structural DA is built.

** Definition 7**. Let PC be a probabilistic classifier with structural description SD. Given an input x∈X and the resulting value ω=PC(C|x) for class *C*∈**C** given **x**, a *LIME/SHAP explanation satisfying structural DA* (*SDA-LIME*/*SDA-SHAP* in short) is an RX $\langle R_{+},R_{-},C,\text{x}\rangle$ such that $R_{+}\cup R_{-}\subseteq \mathrm{SD}$ and

- $R_{+}=\left\{\left(V_{i},V_{j}\right)\in \mathrm{SD}|w\left(\text{x},V_{i},V_{j}\right)>0\right\}$, and
-
$R_{-}=\left\{\left(V_{i},V_{j}\right)\in \mathrm{SD}|w\left(\text{x},V_{i},V_{j}\right)<0\right\}$

where *w* is calculated, resp., using LIME/SHAP iteratively on the sub-classifiers induced by the structural description.

In practice, SDA-LIME and SDA-SHAP result from applying the attribution methods not on “black box” reasons (i.e., explaining class variables in terms of input features alone) but rather on reasons drawn from the structural description. In a nutshell, this amounts to applying LIME and SHAP by following the dependencies included in SD, namely treating parents of class variables as features, in the context of sub-classifiers induced by SD, step-wise. In the first iteration, for each class variable whose parents are all features (note that at least one such variable must exist), LIME and SHAP are applied to the sub-classifier consisting of the variable and its parents, and the weight computed for each parent is assigned to the link from the parent to the variable. Then, for the purposes of the subsequent iterations, each class variable to which this computation has been applied is marked as covered. As a consequence, new variables whose parents are all features or covered will be identified and LIME and SHAP will be applied to the relevant sub-classifiers as above. The process will terminate when reaching the coverage of all variables.

As a simple example, Figure 1ii gives an illustration of the application of SDA-SHAP for the structural description in Table 2c. In the first iteration, SHAP is applied to the sub-classifier consisting of variable *c* (the only one whose parents are all features) and its parents, i.e., to the classifier in Table 2a, giving rise to *w*(**x**, *d, c*) = 0.04, *w*(**x**, *h, c*) = −0.19, *w*(**x**, *n, c*) = 0.18. Then *c* is covered and SHAP can be applied to the classifier consisting of variable *e* and its parents (Table 2b), obtaining *w*(**x**, *s, e*) = −0.19, *w*(**x**, *c, e*) = 0.31 and completing the coverage of the variables.

Note that, like SDA-SHAP, Shapley Flow, recently proposed by Wang et al. (2021), generalizes SHAP so that reasons, rather than feature variables, are assigned a numerical weight. This is done using a causal model as the structural description for features and classes, in order to remove the risk that features not used by the model are assigned non-zero weights. Though featuring a similar high-level goal and sharing some basic idea, Shapley Flow significantly differs from SDA-SHAP. As a first remark, Shapley Flow is limited to single class variables, whereas SDA-SHAP can be used with probabilistic classifiers with any number of class variables. More importantly, in Shapley Flow the weights assigned to edges correspond to a notion of global flow rather than to a notion of importance of local influences, and hence have a different meaning wrt SDA-SHAP.

SDA-LIME and SDA-SHAP of course satisfy structural DA (by design) but fail to satisfy naive and dialectical DA.

** Proposition 4**. SDA-LIME & SDA-SHAP satisfy structural but are not guaranteed to satisfy naive nor dialectical DA.

The results above show that in order to guarantee the satisfaction of all the DA properties, an alternative approach to the construction of bipolar argumentation frameworks for explanation is needed. To this purpose, we introduce the novel *dialectically accurate relational explanations (DARXs)*, whose definition is driven by the set of requirements we have identified.

** Definition 8**. Given a probabilistic classifier with structural description SD, a *dialectically accurate relational explanation* (DARX) is a relational bipolar explanation $\langle R_{+},R_{-},C,\text{x}\rangle$ where, letting $v_{x}=\mathrm{PC}\left(V_{x}|\text{x}\right)$ for any *V*_*x*_∈**V**:

- ${ℛ}_{+}=\{(V_{i},V_{j})\in SD|\forall v_{i}^{\prime }\in \Omega_{V_{i}}\backslash \{v_{i}\}$ it holds that $P(V_{j}=v_{j}|x)>P(V_{j}=v_{j}|x,set(V_{i}=v_{i}^{\prime }))\}$;
- ${ℛ}_{-}=\{(V_{i},V_{j})\in SD|\forall v_{i}^{\prime }\in \Omega_{V_{i}}\backslash \{v_{i}\}$ it holds that $P(V_{j}=v_{j}|x)<P(V_{j}=v_{j}|x,set(V_{i}=v_{i}^{\prime }))\}$.

** Proposition 5**. DARXs are guaranteed to satisfy naive, structural and dialectical DA.

For illustration, suppose SD corresponds exactly to the links in Figure 1iii. Then, this figure shows the DARX for *e* given the input in the figure's caption and the classifier in Table 1 (or Table 2). Here, the satisfaction of naive DA ensures that no spurious reasons, i.e., where the corresponding variables do not, in fact, influence one another, are included in the DARX. Note that, when explaining *e* with the same input, SHAP may draw a positive reason from *d* to *e* (as in Figure 1i) when, according to SD, *d* does not directly affect *e*. Further, the satisfaction of dialectical DA means that each of the reasons in the DARX in Figure 1iii is guaranteed to have the desired dialectical effect (e.g., that the current value of *n* renders the (positive) prediction of *c* more likely, while the value of *h* has the opposite effect). Meanwhile, the RXs (Figures 1i, ii) include the positive reasons from *d*, which have no bearing on either classification for this input.

Note that the bipolar argumentation frameworks representing DARXs are conceived as local explanations, i.e., they are meant to explain the behavior of the classifier given a specific input, not the behavior of the classifier in general. In other words, they assign a positive or negative role to variables with reference to the specific input considered and it may of course be the case that, given a different input, the same variable has a different role.

While DARX provides a notion of local explanation based on bipolar argumentation frameworks which is fully compliant with DA requirements, one may wonder whether its advantages are significant when applied to actual instances of probabilistic classifiers and whether it is viable in terms of performance. These questions are addressed by the empirical evaluation presented in next section.

## 7. Empirical evaluation

As mentioned in Section 4, we experiment with (chains of) BCs as well as chains (in the form of trees) of tree-based classifiers (referred to as *C-DTs* below). As far as BCs are concerned, we experiment with different types, corresponding to different restrictions on the structure of the underlying Bayesian network and conditional dependencies: naive BCs (*NBC*) (Maron and Kuhns, 1960); tree-augmented naive BCs (*TAN*) (Friedman et al., 1997); and *chains* of BCs (Zaragoza et al., 2011), specifically in the form of chains of the unrestricted BCs suggested in Provan and Singh (1995) (*CUBC*). We choose C-DTs and (chains of) BCs because they are naturally equipped with underlying structural descriptions, which allows us to evaluate structural DA, while they are popular methods with tabular data, e.g., in the case of BCs, for medical diagnosis (Lipovetsky, 2020; McLachlan et al., 2020; Stähli et al., 2021).^9^

Our experiments aim to evaluate the satisfaction/violation of structural and dialectical DA empirically for various concrete RXs (i.e., LIME, SHAP and their structural variants) when they are not guaranteed to satisfy the properties, as shown in Section 6.

The main questions we aim to address concern *actual DA* and *efficiency*, as follows. **Actual DA**. While some approaches may not be guaranteed to satisfy DA in general, they may for the most part in practice. *How much DA is achieved in the concrete settings of SHAP, LIME, SDA-SHAP and SDA-LIME explanations?* We checked the average percentages of reasons in LIME and SHAP explanations and in their structural counterparts which do not satisfy our notions of descriptive accuracy. The results are in Table 4. We note that: **(1)** LIME often violates *naive descriptive accuracy*, e.g., in the *Child* and *Insurance* BCs, whereas SDA-LIME, SHAP and SDA-SHAP do not; **(2)** LIME and SHAP systematically violate *structural descriptive accuracy*; **(3)** LIME, SHAP and their structural counterparts often violate *dialectical descriptive accuracy*.

**Table 4:** Average percentages of reasons (over 100 samples) *violating* DA (i.e., $\left|\left\{\left(V_{i},V_{j}\right)\in R_{-}\cup R_{+}\text{such that}\left(V_{i},V_{j}\right)\text{violates DA}\right\}|/|R_{-}\cup R_{+}\right|$) for several instantiated RXs.

**Dataset**	Classifier^*^	**SHAP**			**LIME**			**SDA-SHAP**		**SDA-LIME**	
**Naive**	**Structural**	**Dialectical**	**Naive**	**Structural**	**Dialectical**	**Naive**	**Dialectical**	**Naive**	**Dialectical**
Shuttle	NBC	0%	0%^†^	16.43%	0%	0%^†^	17.14%	‡	‡	‡	‡
German	NBC	0%	0%^†^	54.56%	0%	0%^†^	49.55%	‡	‡	‡	‡
California	TAN	0%	0%^†^	16.75%	0%	0%^†^	16.75%	‡	‡	‡	‡
Insurance	CUBC	0%	67.07%	78.77%	59.56%	89.26%	93.07%	0%	41.77%	0%	42.56%
Child	CUBC	0%	70.97%	75.35%	63.74%	89.59%	91.16%	0%	21.18%	0%	21.18%
HELOC	C-DTs	51.77%	100%	94.42%	62.21%	100%	97.87%	25.60%	77.88%	31.21%	82.09%
LC	C-DTs	16.19%	100%	94.47%	72.95%	100%	98.57%	0%	52.26%	0%	57.63%

(*) NBC (Naive BC), TAN (Tree-Augmented NBC), CUBC (Chain of Unrestricted BCs), C-DTs (Chain of Decision Trees); (†) results must be 0.0% due to the BC type; (‡) SDA-LIME and SDA-SHAP explanations are equal to LIME and SHAP, resp., due to the BC type.

**Efficiency**. We have defined DARXs so that they are guaranteed to satisfy structural and dialectical DA. *Is the enforcement of these properties viable in practice, i.e., how expensive is it to compute DARXs?* Formally, the computational cost for DARXs can be obtained as follows. Let *t*_*p*_ be the time to compute a prediction and its associated posterior probabilities.^10^ The upper bound of the time complexity to compute a DARX is $T_{DARX}\left(\Omega \right)=O\left(t_{p}\cdot \underset{V_{i}\in \text{V}}{\sum }|\Omega_{V_{i}}|\right)$, which is *linear* with respect to the sum of the number of variables' values, making DARXs *efficient*.

## 8. Human experiment

Toward the goal of complying with human-centric requirements for explanations, we introduced *dialectical descriptive accuracy* as a cognitively plausible property supporting trust and fairness but lacking in some popular model-agnostic approaches. We hypothesize that dialectical DA aligns with human judgement. To assess our hypothesis, we conducted experiments on Amazon Mechanical Turk through a Qualtrics questionnaire with 72 participants. Of these, only 40 (56%) passed attention checks consisting of (1) basic questions for trivial information visualized on the screen and (2) timers checking whether the user was skipping very quickly through the questions. We used the Shuttle dataset to test our hypothesis. Indeed, this captures a setting with categorical (not only binary) observations, keeping participants' cognitive load low with an underlying classification problem easily understandable to lay users (see information about user expertise in Appendix 3).

**Figure 2:**
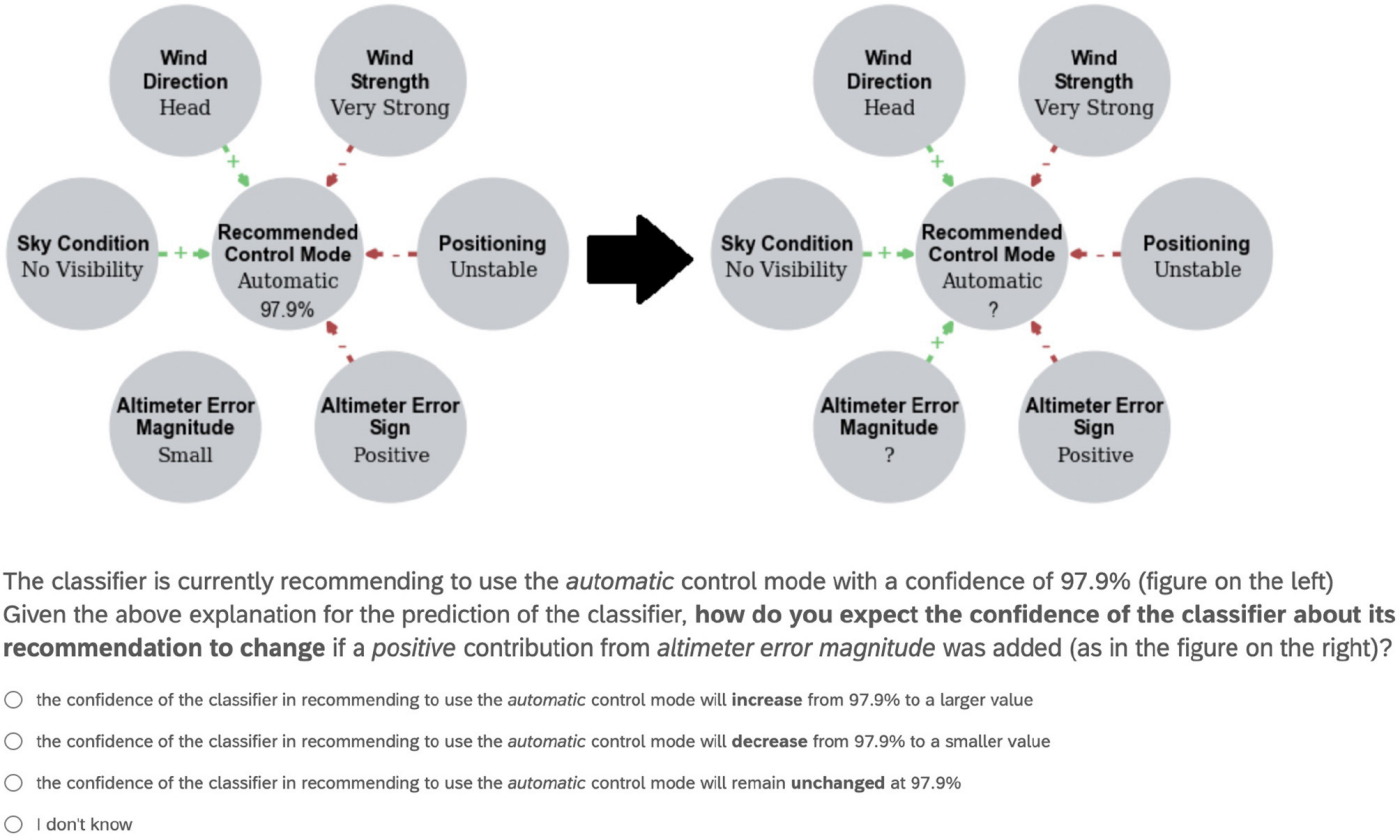
Example question as presented to users in the human experiment.

**Figure 3:**
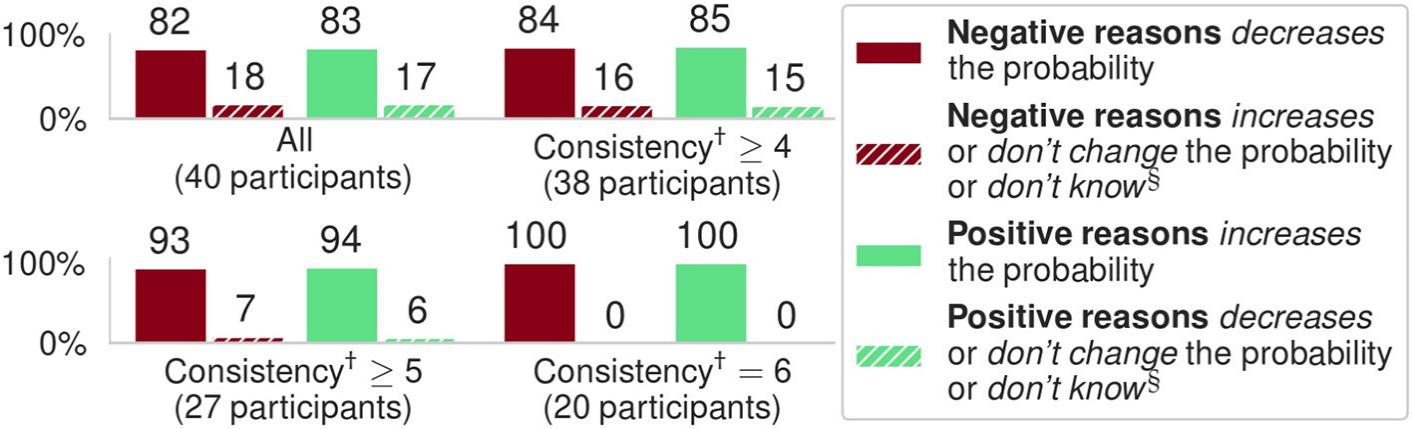
Results of the experiments with 40 participants; all results are significant (*p* << 0.001) against the null hypothesis of random answers. Here, “Negative/Positive reasons” refers to adding negative/positive contributions, resp., from features, as illustrated in Figure 2. (†) *Consistency* represents the number of questions (out of 6) users answered following the same pattern (also unexpected ones, e.g., that negative reasons increase probability). (§) We aggregated all results for unexpected answers in these bar plots.

We presented users with six questions, each accompanied by a DARX in the form exemplified in Figure 2 left, with six feature variables (i.e., *Wind Direction, Wind Strength, Positioning, Altimeter Error Sign, Altimeter Error Magnitude* and *Sky Condition*) assigned to various values and with corresponding predicted probability *p* for the (shown value of the) class variable *Recommended Control Mode*, as computed by our NBC for Shuttle (in Figure 2 left, *p* = 0.979 for the *Automatic* value of the class variable, given the values of the feature variables as shown). The graphical view demonstrated in the figure is a representation of a DARX as defined in Definition 8, with the green/red edges representing the positive/negative resp. reasons. We asked the users how they expected *p* to change when adding a positive (green arrow labeled with +) or negative (red arrow labeled with -) reason, e.g., Figure 2 shows how we asked the users what effect they thought that adding the positive reason *Altimeter Error Magnitude* would have (as in the DARX on the right). Specifically, we asked users to choose among options:

(a) *p* increases;
(b) *p* decreases;
(c) *p* remains unchanged; and
(d) I don't know,

as indicated in Figure 2. For our hypothesis to hold we expected users to select answer (a) when adding positive reasons (as in Figure 3) and to select answer (b) when adding negative reasons. We also assessed how consistent users were, dividing the results based on the number of questions (out of 6) users answered following the same pattern, e.g., consistency of 6 means either all answers aligned with our hypothesis or all answers did not, while consistency of 3 means half of the answers aligned and half did not.

The results are shown in Figure 3: here, when computing the p-values against the null hypothesis of random answers (50%-50%) we used the multinomial statistical test. We note that: **(1)** in all cases users' answers were predominantly in line with our expectations; and **(2)** participants that were consistent in answering more questions were more likely to agree with our hypothesis.

## 9. Conclusions

In this paper we have studied how to define and enforce properties of explanations for the outputs of AI models (focusing on probabilistic classifiers), so that they can be deemed trustworthy and fair, in the sense that they do not mislead their users. Specifically, we have introduced a three-fold notion of DA for explanations of probabilistic classifiers, which, despite its intuitiveness, is often not satisfied by prominent explanation methods, and shown that it can be satisfied, by design, by the novel explanation concept of DARXs. We have performed a wide-ranging evaluation with theoretical results and experiments in a variety of data-centric settings and with humans wrt explanation baselines, highlighting the importance of our most demanding notion of DA (dialectical DA), from a human perspective. This demonstrates how DA, which has thus far been overlooked in the explainable AI literature, could be a critical component in achieving trustworthy and fair systems, in line with the principles of human-centric AI. We have built our definitions of DA and DARX around notions inspired by formal notions of argumentation, thus providing some instantiated evidence about the foundational role of argumentation for human-centric AI, on which the present special issue is focused.

Our work opens several avenues for future work. It would be interesting to experiment with other forms of probabilistic classifiers, including (chained) neural networks, possibly in combination with methods for extracting *causal models* from these classifiers (e.g., as in Kyono et al., 2020) to provide structural descriptions for satisfying structural DA. It would also be interesting to study the satisfaction of (suitable variants of) DA, e.g., those incorporating zero-valued variables as mentioned previously, by other forms of explanations, including *minimum cardinality* explanations (Shih et al., 2018) and set-based explanations (Dhurandhar et al., 2018; Ignatiev et al., 2019). We also note that our proposed methodology in this paper can support human users' full understandings of model intricacies toward leading to their outputs. However, as with other explanation models, there is a fine line between explainability and manipulability. Thus, it would be interesting to explore potential risks in revealing the inner workings of probabilistic classifiers to end users, as this may empower users to manipulate them. We would also like to extend the human experiment described in Section 8 to present a more rigorous assessment of our notions of DA, e.g., assessing structural DA, which would require users who are able to appreciate the model's underpinning structure. Last but not least, while the human experiment provided encouraging indications about the cognitive plausibility of the proposed approach, the present research needs to be complemented by an investigation focused on the Human-Computer Interaction (HCI) aspects involved in properly conveying explanations to users. The fact that HCI principles and methodologies are of paramount importance in human-centric AI has been pointed out by several works in the literature (see e.g., Xu, 2019; Shneiderman, 2020), which also stress the need to properly take into account human and ethical factors. In particular, interactivity is a key factor to address the basic tension between interpretability and accuracy, especially when dealing with complex models (Weld and Bansal, 2019). This is demonstrated, for instance, in case studies where suitable interaction mechanisms are used to allow users to combine global and local explanation paradigms (Hohman et al., 2019) or to enable heuristic cooperation between users and machine in a challenging context like the analysis of complex data in the criminal justice domain (Lettieri et al., 2022).

## Author's note

This paper extends (Albini et al., 2022) in various ways; in particular we introduce novel variants of LIME and SHAP, which satisfy structural DA by design, and we undertake a human experiment examining our approach along the metric of *consistency*.

## Data availability statement

The original contributions presented in the study are included in the article/supplementary material, further inquiries can be directed to the corresponding author.

## Ethics statement

Ethical review and approval was not required for the study on human participants in accordance with the local legislation and institutional requirements. Written informed consent for participation was not required for this study in accordance with the national legislation and the institutional requirements.

## Author contributions

EA had the main responsibility for the implementation and experiments. All authors contributed equally to the conceptual analysis, formal development, and writing of the paper. All authors contributed to the article and approved the submitted version.

## Footnotes

^1^Note that some of these probabilistic classifiers are based on models which are, in principle, interpretable, like Bayesian networks. However we remark that interpretable models may still need explanations (Lipton, 2018; Ciatto et al., 2020; Du et al., 2020), e.g., because their size is beyond human interpretation capabilities or because lay users cannot understand probabilities.

^2^Thus, in this paper we discretise continuous variables, leaving their full treatment to future work.

^3^Our focus is on explaining the outputs of classifiers, so we ignore how they are obtained, e.g., by hand or from data (subject to whichever bias), and how they perform computation.

^4^BCs determine probabilities based on prior and conditional probabilities, e.g., using maximum a posteriori estimation. Given that our focus is on explaining, we ignore here how BCs are obtained.

^5^We stress that what we call here explanations are in fact abstractions of what full-fledged explanations typically are (e.g., feature attribution methods such as LIME (Ribeiro et al., 2016) and SHAP (Lundberg and Lee, 2017) include, in addition to positive and negative features, a numerical value therefor). In this sense, we could more appropriately refer to our abstractions as *explanation skeletons*, but refrain to do so for simplicity of exposition.

^6^All proofs not included in the paper can be found in Appendix 1.

^7^We omit the formal definitions of how these well-known methods compute *w*, as the considerations in this paper on property satisfaction for LIME and SHAP are mostly based on empirical evaluation, supported by the standard implementations of LIME and SHAP, rather than their formal definition.

^8^While our property of naive DA requires that features included in an explanation are, in fact, relevant, a dual requirement would be that features not included in an explanation are, in fact, irrelevant. Addressing also this requirement corresponds to considering the implicit contents of an explanation too, i.e., all the features which are not presented to the user. However, the choice of features to be presented to the users may be determined also by their importance degree. In this sense, a feature which is not included does not necessarily need to be completely irrelevant, while a feature which is included definitely needs to be relevant. Based on this considerations, in this paper we focus on naive DA as a fundamental requirement, while we leave the investigation of more articulated versions of this property to future work.

^9^We use several datasets or (pre-computed) Bayesian networks, and deploy the best-performing type of the chosen type of classifier for each. Dataset sources were as follows: Shuttle (UCI, 2020), German (UCI, 2020), California (Kelley Pace and Barry, 1997), Child (BNlearn, 2020), Insurance (BNlearn, 2020), HELOC (Community, 2019) and LendingClub (LC) (Club, 2019). As structural descriptions, we use those described in Section 4. When training classifiers on datasets, we split them into train/test sets and optimize the hyper-parameters. See Appendix 2 for details on the datasets, training and performance, and for further details on the experiments.

^10^In our experiments, using a machine with *Intel i9-9900X* at 3.5*Ghz* and 32*GB* of RAM with no GPU acceleration, *t*_*p*_ ranges from 3μ*s* for the simplest NBC to 436*ms* for the most complex chain classifier.
