# Supplementary material for: Achieving descriptive accuracy in explanations via argumentation: The case of probabilistic classifiers
Source: Front Artif Intell. 2023 Apr 6;6:1099407. doi: 10.3389/frai.2023.1099407 (PMC10117939; doi:10.3389/frai.2023.1099407)
Supplement: Supplementary file 1 [file Data_Sheet_1.pdf]

Achieving Descriptive Accuracy in Explanations via Argumentation: the Case of Probabilistic Classifiers - Supplementary Material

Emanuele Albini [1], Antonio Rago [1,*], Pietro Baroni [2] and Francesca Toni [1]

[1] *Department of Computing, Imperial College London, UK*
[2] *Dipartimento di Ingegneria dell'Informazione, Università degli Studi di Brescia, Italy*

Correspondence*:
Corresponding Author
a.rago@imperial.ac.uk

1 PROOFS

Here we provide the proofs for the propositions and claims from the paper (we re-include the statements for readability).

PROPOSITION 1. *If a bipolar explanation $\langle \mathbf{F}_+, \mathbf{F}_-, C, \boldsymbol{x} \rangle$ satisfies dialectical DA then it satisfies naive DA.*

PROOF. If a bipolar explanation $\langle \mathbf{F}_+, \mathbf{F}_-, C, \mathbf{x} \rangle$ satisfies dialectical DA for an input $\mathbf{x}$ then:

- If $\mathbf{F}_+ \cup \mathbf{F}_- = \varnothing$ then $\langle \mathbf{F}_+ \cup \mathbf{F}_-, C, \mathbf{x} \rangle$ satisfies naive DA trivially.
- If $\mathbf{F}_+ \cup \mathbf{F}_- \neq \varnothing$, then by inspection of Property 2, for every $X_i \in \mathbf{F}_+ \cup \mathbf{F}_-$, for every $\mathbf{x}' \in \mathcal{X}$ with $\mathbf{x}'(X_j) = \mathbf{x}(X_j)$ for all $X_j \neq X_i$ and $\mathbf{x}'(X_i) \neq \mathbf{x}(X_i)$, letting $\omega = \mathcal{PC}(C|\mathbf{x})$, it holds that if $X_i \in \mathbf{F}_+$ then $P(C = \omega|\mathbf{x}) > P(C = \omega|\mathbf{x}')$, and thus $P(C = \omega|\mathbf{x}) \neq P(C = \omega|\mathbf{x}')$; if $X_i \in \mathbf{F}_-$ then $P(C = \omega|\mathbf{x}) < P(C = \omega|\mathbf{x}')$, and thus $P(C = \omega|\mathbf{x}) \neq P(C = \omega|\mathbf{x}')$. Thus, the unipolar explanation satisfies naive DA.

PROPOSITION 2. *If an RX $\langle \mathcal{R}_+, \mathcal{R}_-, C, \boldsymbol{x} \rangle$ satisfies dialectical DA then it satisfies naive DA.*

PROOF. The proof has the same structure as that of Proposition 1, but uses pairs instead of features.

PROPOSITION 3. *In general, LIME and SHAP explanations are not guaranteed to satisfy naive nor dialectical DA.*

PROOF. The classifier in Table 1 in the main body gives a counterexample where SHAP does not satisfy naive nor dialectical DA. Given that LIME is an empirical method based on sampling, to assess whether it satisfies naive DA we added a *spurious feature* to the German NBC used in Section 7 and a NBC we built using the COMPAS dataset from the ProRepublica Data Store (ProPublica, 2016). This spurious feature was randomly generated and ignored by the BCs. Table 1 (below), first column, shows the percentages of LIME explanations using this spurious feature in positive or negative reasons. Each of them represent a counterexample of naive DA, thus proving the result. The second column shows the same percentage when using the default, approximate implementation of SHAP on the same BCs. We did this additional experiment to give evidence of the practical relevance of the violations of DA in the actual uses of SHAP. Since naive DA is not guaranteed, by Proposition 2, neither is dialectical DA.

Dataset	LIME	SHAP
**COMPAS**	100%	59.92%
**German**	100%	1.31%

Table 1. Percentages of LIME/SHAP using a spurious feature (thus violating naive DA, and thus dialectical DA) in a positive or negative reason for the COMPAS and German BCs.

PROPOSITION 4. *SDA-LIME & SDA-SHAP satisfy structural but are not guaranteed to satisfy naive nor dialectical DA.*

PROOF. Structural DA is satisfied by construction (as SDA-LIME and SDA-SHAP are defined so that they satisfy this property). To assess whether SDA-SHAP and SDA-LIME satisfy naive DA, we look at the counterexamples from the proof for Proposition 3. Since German is an NBC, the SDA-LIME and SDA-SHAP explanations are equivalent to the LIME and SHAP explanations (resp.) and these counterexamples hold for the SDA versions of the explanations. Thus, SDA-LIME and SDA-SHAP do not satisfy naive DA and so, by Proposition 2, they do not satisfy dialectical DA.

PROPOSITION 5. *DARXs are guaranteed to satisfy naive, structural and dialectical DA.*

PROOF. Satisfaction of dialectical and structural DA is by definition. Satisfaction of naive DA follows from Proposition 2.

2 EMPIRICAL EXPERIMENTS SETUP

We will provide all the code and data upon publication.

Dataset	Type*†	Size			Variables‡		Performance	
		Instances	$\lvert O\rvert$	$\lvert C\rvert$	O	C	Accuracy	F1
**Shuttle**	NBC, DS	278	6	1	C	B	95.7%	0.96
**German**	NBC, DS	750	20	1	C	B	76.4%	0.72
**California**	TAN, DS	20433	12	1	C	C	81.0%	0.80
**Child**	CUBC, BN	1080	7	13	C	C	–¶	–¶
**Insurance**	CUBC, BN	1500	8	19	C	C	–¶	–¶
**HELOC**	C-DTs, DS	10459	23	11	C	B	0.61	0.60
**LC**	C-DTs, DS	100000◊	19	12	C	B	0.80	0.88
**COMPAS**	NBC, DS	6951	12	1	C	B	70.5%	0.71

Table 2. Characteristics of the datasets used in the empirical evaluation are in the proofs of our theoretical results. (∗) NBC (Naive BC), TAN (Tree-Augmented NBC), CUBC (Chain of Unrestricted BCs), C-DTs (Chains of Decision Trees); (†) **D**ata**S**et (DS) or **B**ayesian **N**etwork (BN); (‡) **B**inary or **C**ategorical; accuracy and macro F1 score on the test set, averaged for multi-label settings; (¶) no dataset was provided (a Bayesian network was given directly instead) to test performance; (◊) Given the sheer size of the dataset, we took a random sample of 100,000 samples.

2.1 Datasets

Table 2 reports details of the settings for the experiments we ran (note that COMPAS is used for the proof of Proposition 3). We divided the datasets into randomly stratified train and test sets with a 75/25% split. When the data source was a Bayesian network, in order to generate the test set for the experiments we

artificially generated the combinatorial dataset with all possible combinations of inputs and calculated the classifications using variable elimination (an exact inference algorithm for Bayesian networks).

2.2 Model Training

Table 2 summarises the model type and the software implementation that we used for each setting we experimented with. We note that for all settings in which an (already trained) Bayesian network was not given, we trained a model. We will now detail the hyper-parameters in each setting.

Shuttle. We trained an `sklearn` `CategoricalNB` classifier with Laplace smoothing coefficient $\alpha = 5$ and classes prior probabilities $\beta = \langle 0.8, 0.2 \rangle$. The model hyper-parameters were optimised using random grid search under a 5-fold cross validation.

German. In order to train a discrete classifier, we discretized continuous features using bins with the "same length" (i.e. each bin covers an interval of the same length). In particular, we used 10 bins for the feature *age* and 9 bins for features *amount* and *duration*. We then trained an `sklearn` `CategoricalNB` classifier with Laplace smoothing coefficient $\alpha = 0.00001$ and classes prior probabilities $\beta = \langle 0.35, 0.65 \rangle$. The model hyper-parameters were optimised using random grid search under a 5-fold cross validation.

California. We trained an `sklearn` `CategoricalNB` classifier with Laplace smoothing coefficient $\alpha = 2$ and classes prior probabilities $\beta = auto$ (we used the default setting of sklearn based on automatically computing the classes priors' based on their prevalence in the training data). The model hyper-parameters were optimised using random grid search under a 5-fold cross validation. We used a custom strategy to discretize continuous features for this dataset because it was performing better than automatic ones.

COMPAS. We trained an `sklearn` `CategoricalNB` classifier with Laplace smoothing coefficient $\alpha = 0.1$ and classes prior probabilities $\beta = auto$. The model hyper-parameters were optimised using random grid search under a 5-fold cross validation. We used a custom strategy to discretize continuous features because it was performing better than automatic ones.

Child and **Insurance**. A Bayesian network was given in these settings. We loaded the models using `pgmpy` `BayesianModel` (all probabilities are given in the Bayesian network, they do not need to be learnt).

HELOC. Similarly to (Rudin, 2019) we used 10 disjoint subsets of the features in order to predict 10 intermediate-level classifications: *ExternalRisk*, *TradeOpenTime*, *SatisfactoryTrades*, *TradeFrequency*, *Delinquency*, *Installment*, *Inquiry*, *RevolvingBalance*, *Utilization*, *TradeWBalance*. This 10 intermediate-level classifications are then used to predict the target class: *RiskPerformance*. In practice, we trained 10 `skelarn` `DecisionTreeClassifier` models for each of the intermediate-level classifications that are then chained together as features of a final `skelarn` `DecisionTreeClassifier` trained to predict the target class. We used the default hyper-paramenters of `DecisionTreeClassifier`. We discretized continuous features following the same dicretization procedure proposed in (Rudin, 2019).

LC. Similarly to HELOC, we used 11 disjoint subsets of the features in order to train 10 intermediate-level `sklearn` `DecisionTreeClassifier` (*Amount*, *Repayment*, *Application*, *Applicant*, *Delinquency*, *Number*, *Utilization*, *Balance*, *Age*, *Home*, *Grade*) that were then chained together to train another `sklearn` `DecisionTreeClassifier` trained to predict the target: *loan_status*. For this dataset, we discretized continuous features using same size bins (i.e. each bin has the same number of elements from the dataset).

2.3 Experiments Setup

To run our experiments, we used a machine with Ubuntu 18.04, an Intel processor with 10 cores running at 3.5Ghz, 32GB of RAM and no GPU acceleration.

The experiments on satisfaction of properties by the explanations were run on (all the samples of) the test set, and if the number of samples in the test set was greater than 100 samples, we ran them on a random sample of 100 samples. LIME explanations were generated using the `lime 0.1.1.37` package [1] with default parameters. SHAP explanations were generated using the `shap 0.35.0` package [2] with default parameters. For all the random computations (`sklearn`, `pandas`, `numpy`, `random`) we used a random seed of 0. We used `pgmpy 0.1.6` and `scikit-learn 0.22.2` in a Python 3.6 environment to train the models.

3 HUMAN EXPERIMENTS: EXPERTISE

Almost all of the participants were native English speakers, with three being fluent (C1-C2). The highest level of education was high school in twelve participants, while the majority had Bachelor's degrees. With regards to the participants expertise, some level of expertise in computer science but the majority did not. The levels of expertise in machine learning, mathematics and statistics were slightly lower. Meanwhile, very few had expertise with Bayesian classifiers or familiarity with LIME or SHAP.

4 EXPLANATION GENERATION RUNTIME

Dataset	Classifier*†	DARX	SHAP	LIME	SDA-SHAP	SDA-LIME
**Shuttle**	NBC, DS	2ms	22ms	58ms	‡	‡
**German**	NBC, DS	6ms	471ms	18ms	‡	‡
**California**	TAN, DS	458ms	2,127,452ms	103,083 ms	‡	‡
**Insurance**	CUBC, BN	1,076ms	1,370ms	508ms	530ms	639ms
**Child**	CUBC, BN	511ms	7,734	4,162ms	386ms	456ms
**HELOC**	C-DTs, DS	700ms	22,000 ms	1,700ms	1,144ms	889ms
**LC**	C-DTs, DS	1,290ms	24,777ms	1,692ms	10,501ms	571ms

Table 3. Average runtime to generate an explanation (over 100 samples) (∗) NBC (Naive BC), TAN (Tree-Augmented NBC), CUBC (Chain of Unrestricted BCs), C-DTs (Chains of Decision Trees); (†) **D**ata**S**et (DS) or **B**ayesian **N**etwork (BN); (‡) **B**inary or **C**ategorical; (‡) SDA-LIME and SDA-SHAP explanations are equal to LIME and SHAP, resp., due to the BC type.

REFERENCES

[Dataset] ProPublica, D. S. (2016). Compas recidivism risk score data and analysis

Rudin, C. (2019). Stop explaining black box machine learning models for high stakes decisions and use interpretable models instead. *Nature Machine Intelligence* 1, 206

[1] https://github.com/marcotcr/lime

[2] https://github.com/slundberg/shap
